# Complete Genome Sequence of *Mycoplasma ovis* Strain Michigan, a Hemoplasma of Sheep with Two Distinct 16S rRNA Genes

**DOI:** 10.1128/genomeA.01235-13

**Published:** 2014-01-30

**Authors:** Pierre L. Deshuillers, Andrea P. Santos, Naíla C. do Nascimento, Joseph A. Hampel, Ingrid L. Bergin, Melissa C. Dyson, Joanne B. Messick

**Affiliations:** a Department of Comparative Pathobiology, Purdue University, West Lafayette, Indiana, USA; b Unit for Laboratory Animal Medicine, University of Michigan Medical School, Ann Arbor, Michigan, USA

## Abstract

We report the complete genome sequence of *Mycoplasma ovis* strain Michigan. Its single circular chromosome has 702,511 bp and contains 2 copies of the 16S rRNA gene, one corresponding to *M. ovis* and the other to “*Candidatus* Mycoplasma haemovis.” All housekeeping genes and the 5S-23S rRNA genes are present in single copies.

## GENOME ANNOUNCEMENT

*Mycoplasma ovis* (*Eperythrozoon ovis*), a hemoplasma infecting sheep, was first described by Neitz et al. in 1934 (1). While acute infection in sheep may result in anemia and ill thrift syndrome, most animals do not show clinical signs of infection. *M. ovis* infection has also been described in goats, deer, and reindeer (2–7).

A second genetically distinct hemoplasma of sheep and goats was detected by cloning and sequencing of its 16S rRNA genes (8). In addition to a missing stretch of 17 nucleotides and an overall sequence identity of only 97% compared to *M. ovis*, the pathogenicity of this strain or species is possibly different (8). Subsequent studies identified this novel hemoplasma as “*Candidatus* Mycoplasma haemovis” (3, 9, 10).

Genomic DNA from *M. ovis* strain Michigan was purified from blood of a naturally infected sheep (11, 12) using the Quick-gDNA Blood MidiPrep (Zymo Research Corporation, Irvine, CA), according to the manufacturer’s recommendations. The whole genome was sequenced using Illumina HiSeq 2000 (Illumina, Inc., San Diego, CA) and Illumina MiSeq platforms at the Purdue University Genomics Core Facility. Average reads of about 100 bases for Illumina HiSeq 2000 and 250 bases for Illumina MiSeq were assembled using ABySS 1.2.7. After assembly resulting from genome coverages of 1,500× and 1,000× for Illumina HiSeq and Illumina MiSeq, respectively, 3 remaining gaps were closed using conventional PCR, followed by Sanger sequencing in both directions. First-pass annotation was achieved using the Manatee and the NCBI annotation pipelines.

The complete genome of *M. ovis* Michigan consists of 702,511 bp in a single circular chromosome, with an average G+C content of 31.7%. Its general genomic features are similar to those of other hemoplasmas (13–21), with one remarkable exception, the presence of two copies of the 16S rRNA genes corresponding to sequences previously reported for *M. ovis* and “*Candidatus* Mycoplasma haemovis.” Each copy is in a different operon, both separated from the single copy of the 5S-23S rRNA operon. Thirty-two tRNAs were identified, covering all amino acids. A total of 801 protein-coding sequences (CDSs) were predicted. While 323 CDSs have putative functional identities, most (59.7%) of the CDSs encode hypothetical proteins. Like those of other sequenced hemoplasmas, most of the proteins with identified functions are related to energy metabolism and are restricted to the glycolytic pathway and ATP synthesis. The proportion of the genome of *M. ovis* dedicated to duplicated genes organized into paralog families is 32.4%.

This is the first genome sequencing of *M. ovis* and the first hemoplasma reported as having two different copies of the 16S rRNA genes. Further investigations are needed to determine whether *M. ovis* and “*Candidatus* Mycoplasma haemovis” may also exist as separate strains/species or only as a single species with two copies of the 16S rRNA gene.

### Nucleotide sequence accession number.

The genome sequence of *M. ovis* Michigan has been deposited in GenBank under the accession no. CP006935.
